# Total fluid intake assessed with a 7-day fluid record versus a 24-h dietary recall: a crossover study in Indonesian adolescents and adults

**DOI:** 10.1007/s00394-015-0954-6

**Published:** 2015-06-14

**Authors:** Saptawati Bardosono, Romain Monrozier, Inge Permadhi, Nurul Ratna Mutu Manikam, Rizki Pohan, Isabelle Guelinckx

**Affiliations:** Department of Nutrition, Faculty of Medicine, Universitas Indonesia, Ilmu Gizi Lantai 2, Jalan Salemba Raya No. 6, Jakarta, 10430 Indonesia; Hydration & Health Department, Danone Research, Palaiseau, France; R&D AQUA Group, Jakarta, Indonesia

**Keywords:** Water intake, Total fluid intake, 7-day fluid record, 24-h dietary recall, Indonesia

## Abstract

**Purpose:**

To compare total fluid intake (TFI), defined as the sum of water and all other fluid types, assessed with a 24-h dietary (food and fluid) recall with mean TFI assessed with a 7-day fluid-specific record among adolescents and adults.

**Methods:**

This repeated cross-sectional study compared TFI as assessed by two fluid assessment instruments using a crossover approach. 290 adolescents (17.3 ± 0.8 years, 50 % boys) and 289 adults (43 ± 9.3 years, 50 % men) from Indonesia completed the study.

**Results:**

Significant correlations were observed between fluid intake assessed with the 24-h recall and the 7-day fluid record (*r* = 0.333; *p* < 0.001). The Bland–Altman method, however, showed an underestimation (bias) of mean TFI by a 24-h recall when compared with the 7-day fluid record [mean difference (95 % CI) −382 mL (−299, −465); *p* < 0.001]. The mean difference also increased with increasing TFI: Mean difference for the lowest and highest quartiles of TFI was 139 versus −1265 mL/day. The 7-day fluid record recorded two (95 % CI −1.9, −2.4; *p* < 0.0001) extra drinking acts compared with the 24-h recall, whereas the mean volume per drinking act was significantly higher with the 24-h recall [mean difference (95 % CI) 39 mL (31, 47); *p* < 0.001].

**Conclusion:**

Compared with a 7-day fluid record, a 24-h dietary recall significantly underestimated TFI. Subjects recalled two less drinking acts, while estimating the volume consumed per drinking act to be larger. Since the adequate intakes for total water intake are based on median intakes observed in national surveys that most frequently used the 24-h recall method, they may potentially be underestimated.

## Introduction

It is widely known that the composition of the human body is mainly water, and that water is vital for normal body function [1, 2]. Water also plays a role in the maintenance of normal thermoregulation and normal physical and cognitive functions [3]. Therefore, the Indonesian Food and Nutrition Conference 2012 (*Widyakarya Nasional Pangan dan Gizi 2012*) set adequate total daily water intake recommendations (RDI) for adolescent aged 16–18 years at 2.1 L/day for girls and 2.2 L/day for boys, while for adults aged 19–64 years, consuming 2.3 and 2.5–2.6 L/day was recommended for women and men, respectively [4]. The intake of the nutrient water is provided by both foods and fluids (drinking water and beverages of all kind), but mainly by fluids (approximately 80 %).

Surveying populations once recommendations have been made, allows establishing whether or not the recommendations are being met. However, although it is essential to measure water intake from both foods and beverages, until now, no dietary methods have been validated to accurately and precisely estimate total water or total fluid intake (TFI) [1]. Since all instruments have specific limitations, the results obtained from the same respondents, but with different instruments, can *vary *[5]. This may lead to inconsistent conclusions and make comparisons between surveys using different instruments difficult, if not invalid. Therefore, it would be valuable to compare and determine the difference in TFI measured with frequently used methods. A 24-h recall record is a retrospective method that assesses what the respondent consumed during the previous 24 h. It is a relatively quick and inexpensive method, and therefore, it is commonly used in epidemiological surveys. However, this method tends to overestimate “healthy intakes” and underestimate “unhealthy” intakes as intakes are self-reported; in addition, it does not take into account the variability in intakes [5]. On the contrary, a dietary record is a prospective method usually recording the intakes over several consecutive days. Even though this instrument is resource-intensive, time-consuming, and can be a burden on some subjects, it is considered to provide relatively accurate data concerning intake of food and nutrients [6]. Consequently, a 7-day dietary record is used as a criterion method, and as such, other nutrition assessment methods are often compared with it [6]. Recently, a dietary record has been developed to specifically estimate fluid intake over 7 consecutive days in several cross-sectional surveys in different countries from different continents [7, 8]. The aim of the present study was to compare habitual TFI assessed with this 7-day fluid-specific record and with a 24-h dietary recall among apparently healthy Indonesian adolescents (16–18 years) and adults (19–64 years).

## Methods

### Study design

**Fig. 1:**
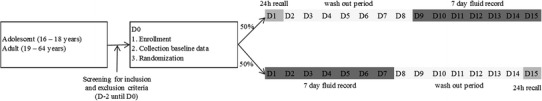
Study with crossover design to assess fluid intake of adolescents and adults with a 24-h dietary recall and a 7-day fluid record

A crossover study design was used to assess fluid intake of the subjects using 24-h recall and 7-day records as shown in Fig. 1; a washout period of 7 days was incorporated into the study design. After screening for inclusion and exclusion criteria, eligible subjects were enrolled, and data on their sociodemographic characteristics and medical history were collected. Physical and anthropometry assessments were also taken. Subjects were randomly assigned to one of the two groups using concealed envelopes. Group 1 started on day 1 with the 24-h recall followed by 7 days of washout period and consequently started on study day 9 with the 7-day fluid diary. The second group started on day 1 with the 7-day fluid record, on day 8 with the washout period, and finished on day 15 with the 24-h dietary recall. Subjects were requested to keep their habitual food and fluid intake throughout the study period of 15 days.

After explaining the study protocol to all subjects, a written informed consent was obtained. The study was approved on April 17, 2012 by the Ethics Committee of the Faculty of Medicine, Universitas Indonesia (number 151/PT02.FK/ETIK/2012).

### Subjects

Two age groups were defined: adolescents aged 16–18 years and adults aged 19–64 years. The study aimed to recruit 300 subjects per age group and 60 subjects per municipality. The municipalities were selected in five areas of Jakarta (North, West, Central, East, and South Jakarta), and from each municipality, one primary health center (*Puskesmas*) was selected to be the study center. Each primary health center identified households that contained at least one adolescent and one adult living together. From these households, all eligible subjects were recruited until the target sample size of this study was reached. Study inclusion criteria were female and male individuals aged 16–64 years, who were apparently healthy based upon a physical examination performed by a physician. Additionally, the subjects had to be resident in the study area for at least 1 year and had to be of middle-level socioeconomic status (B and C based on AC Nielsen criteria [9]). Pregnant women, lactating women, subjects with special dietary restrictions, and subjects who were illiterate and/or had difficulties to communicate orally were not eligible for recruitment.

### Intake assessment methods

#### 24-h dietary recall

Trained nutritionists visited the subjects at home to recall their food and fluid intake during the previous 24-h period. The face-to-face interview was done in four stages. During the first phase, the interviewer obtained a complete list of all foods and beverages consumed the previous day, followed in the immediate second phase by a detailed description of each food and beverage consumed, including cooking methods and brand name. In the third phase, estimates of the amount of each food and beverage item consumed were recorded. Photographs, household utensils, or food models were used as memory aids to assess portion sizes. In the fourth pass, the interviewer repeated all detailed food and beverage items recorded to ensure completeness. Only the data on fluid intake, defined as the intake (in mL) of water and all other beverages, were extracted from the 24-h recall for this analysis.

#### 7-day fluid record

A trained nutritionist delivered and explained the fluid record to the subjects during a face-to-face interview in their homes. Each day, the same nutritionist visited the subject at home to collect the fluid record of the previous day and to provide a new record for the next day. The aim of these daily home visits was to maintain a high participation rate and to avoid subjects copying the previous day’s data into the next-day record. In total, 30 trained nutritionists were involved in the data collection. Each nutritionist was responsible for visiting a maximum of five households or 10 subjects during the same period.

The fluid record was structured to collect the following detailed information on each drinking act in open spaces on the record: the hour of consumption, the type of fluid, the brand of fluid, the volume of the recipient from which the volume was consumed, and the volume actually consumed. For these variables, no answers were proposed to the subjects. For the last two variables, the temperature of the fluid and the location where the drinking act took place, the answers were predefined for the subjects. The temperature at which the fluid was consumed could be: chilled with ice cubes, chilled without ice cubes, warm, or at room temperature. The predefined locations were at home, the office, school/college, a restaurant or canteen, or other location. To assist the subjects in estimating the consumed volumes, the records were supported by a photographic booklet of standard containers of fluids.

### Classification of fluid types

All fluids recorded by the 24-h dietary recall and the 7-day fluid record were classified accordingly: water (bottled water and boiled water), hot beverages (coffee and tea), milk and derivatives, soft drinks (carbonated and non-carbonated sugar-sweetened drinks, carbonated and non-carbonated non-calorically sweetened drinks, ice-based, coconut-based, chocolate-based, and fruit- and vegetable-based drinks), and other beverages (traditional drinks, cereal drinks, herbal drink, soy bean milk, others). TFI was established by the sum of all these categories. Any addition (e.g., sugar) to a fluid was not taken into account during the fluid classification. A drinking act was defined as any act of consumption of any fluid type at any time of the day. Mean volume per drinking act was calculated by dividing TFI by the number of drinking acts.

### Data management and analysis

Data were recorded daily using specific forms, then checked, coded, and entered into spreadsheets (SPSS version 20, SPSS Inc., Chicago, IL). Subjects reporting a mean total daily fluid intake below 0.4 L/day or higher than 6 L/day, as well as subjects not completing all 7 days of the 7-day fluid record were excluded from the analysis. The null hypothesis to test was that both assessment methods did not result in a different TFI (i.e., zero difference), assuming that each individual ate and drank the same way throughout the study period. Within-person intakes of fluids were compared using a general linear model for repeated measured data and Spearman rank correlation test. The Bland–Altman method was used to assess the agreement between the results obtained with both instruments [10]. For each subject, the following values were calculated based on TFI obtained by the 24-h dietary recall and the 7-day fluid record: difference in TFI between both methods (formula 1) and mean TFI for both methods (formula 2). The limits of agreement (LOA, formula 3) were calculated for the total study sample, the adolescent and the adult sample.

1 $${\text{Difference}}_{\text{both methods}} = {\text{TFI}}_{{24{\text{h recall}}}}\,{-}\,{\text{TFI}}_{{7{\text{ day fluid record}}}}$$

2 $${\text{Mean difference}}_{\text{both methods}} = {{\left( {{\text{TFI}}_{{24{\text{h recall}}}} {-}{\text{TFI}}_{{7{\text{ day fluid record}}}} } \right)} \mathord{\left/ {\vphantom {{\left( {{\text{TFI}}_{{24{\text{h recall}}}}\,{-}\,{\text{TFI}}_{{7{\text{ day fluid record}}}} } \right)} 2}} \right. \kern-0pt} 2}$$

3 $${\text{LOA}} = {\text{mean difference}}_{\text{both methods}} \pm 1.96 \times {\text{SD}}_{\text{difference}}$$

with SD_difference_ being the standard deviation of the difference in TFI of both methods.

The Bland–Altman index (%) was calculated as a percentage of intakes beyond LOA. Good reproducibility of the measurement is proven by a minimum of 95 % difference within the ±2SD limits, which corresponds to a Bland–Altman index of no more than 5 %. Additionally, a relative agreement between both dietary methods was also assessed as follows: The TFI obtained with both records was used to classify an individual into adequacy percentage categories (≤50, 50–75, 75–100 or >100 % of RDI for water) of achievement of the Indonesian recommended water intake [4]. Then, a cross-classification was performed to estimate the percentage of subjects classified by the two methods into the same category (agreement), the same plus adjacent category (classified into the same or adjacent category), classified two categories apart (disagreement), and classified into extreme categories (extreme disagreement). A probability (*p*) value of <0.05 was considered statistically significant.

## Results

**Fig. 2:**
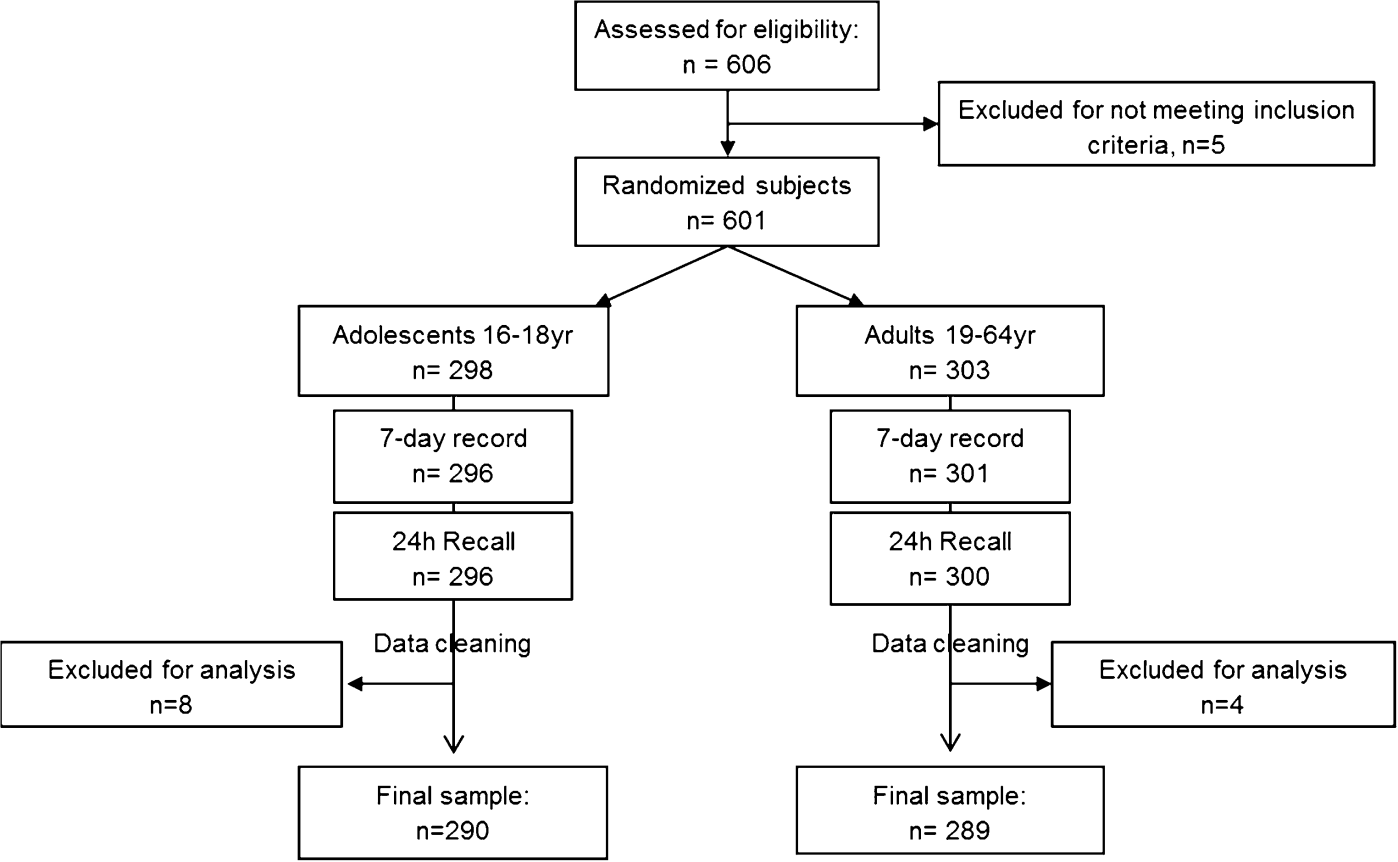
Diagram flow of the study

The subject flow is presented in Fig. 2. Of the 606 eligible subjects, five subjects were not enrolled as they did not meet the inclusion criteria. Of the 298 adolescent subjects, 296 (99 %) completed the 7-day consecutive fluid record and 24-h dietary recall. Among the 303 adult subjects, 301 (99 %) completed both the 7-day consecutive fluid record and 24-h dietary recall. The self-reported baseline demographic characteristics of the final study are shown in Table 1. No significant gender differences in baseline characteristics were observed (data not shown).

**Table 1:** Baseline characteristics of subjects

Variables	Adolescents *n* = 290	Adults *n* = 289
Gender (male/female)	141 (50)/149 (50)	142 (50)/147 (50)
Age (years)	17.3 (0.8)	43.0 (9.3)
Body mass index (BMI) (kg/m^2^)	20.5 (4.0)	25.0 (4.6)
BMI classification^a^		
Thinness	48 (17)	18 (6)
Normal	199 (69)	77 (27)
Overweight	28 (10)	59 (20)
Obese	15 (5)	135 (47)

Data are presented as mean (SD) or n (%)

^a^Body mass index classification following WHO guidelines [31]

**Fig. 3:**
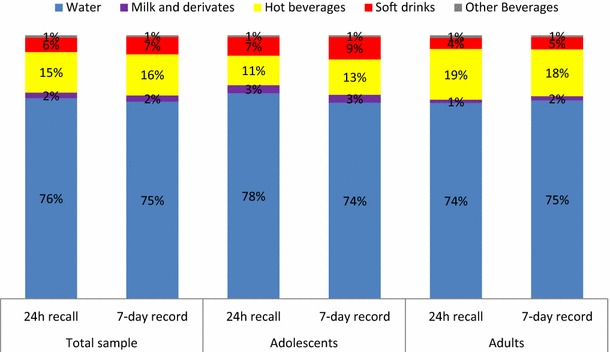
Contribution (%) of the different beverage types to total fluid intake according to the assessment method (24-h dietary recall vs. 7-day fluid record) and age group

Mean TFI estimated with the 24-h dietary recall, mean TFI of the seven individual days, and the mean TFI across the 7 days estimated with the 7-day fluid record are presented in Table 2. Mean TFI showed a wide range for both methods: 500–5440 mL/day for the 24-h recall and 586–5979 mL/day for the 7-day record. The day-to-day fluid intake varied by approximately 50 mL in the 7-day consecutive fluid record data but was not shown to be significant according to the repeated measures GLM multivariate statistical test. Figure 3 presents the contribution of the different fluid types to TFI. The contribution of the different beverage types to TFI estimated by both instruments was comparable for both adolescents and adults.

**Table 2:** Total fluid intake estimated with the 24-h dietary recall and the 7-day fluid record stratified by age group (mL/day)

	Adolescents *n* = 290							Adults *n* = 289						
	Mean	SD	Percentiles					Mean	SD	Percentiles				
			5	25	50	75	95			5	25	50	75	95
24-h dietary recall	1982	786	890	1400	1850	2500	3545	2164	931	960	1440	1900	2760	3793
7-day fluid record														
Day 1	2362	1003	1106	1665	2195	2803	4264	2546	1008	1200	1885	2345	3080	4643
Day 2	2414	1026	1113	1760	2215	2928	4367	2534	1022	1203	1785	2380	3075	4460
Day 3	2375	994	1000	1658	2248	2928	4032	2563	991	1170	1900	2410	3110	4395
Day 4	2377	923	1091	1700	2278	2858	4080	2508	1034	1180	1803	2310	3015	4665
Day 5	2392	1017	1000	1688	2200	2945	4318	2523	968	1200	1845	2330	3000	4340
Day 6	2427	1074	1100	1729	2245	2870	4500	2539	961	1200	1828	2400	3120	4400
Day 7	2398	1010	1100	1750	2240	2825	4114	2490	946	1200	1865	2360	2960	4335
Mean	2392	855	1219	1847	2247	2804	4000	2529	864	1366	1959	2334	3068	4141

The mean TFI of the total sample estimated with the 7-day fluid record was significantly higher than TFI estimated by 24-h dietary recall [mean difference (bias) 382 mL/day; *p* < 0.0001] (Table 3). This was observed for both adolescents and adults (Table 3). Furthermore, two extra drinking acts were reported for the 7-day fluid record compared with the 24-h dietary recall. The estimated volume consumed per drinking act was higher for the 24-h dietary recall than with the 7-day fluid record.

**Table 3:** Total fluid intake (mL/day), mean number of drinking acts, and mean volume per drinking act recorded with the 24-h dietary recall and the 7-day fluid record by adolescents and adults

	Total sample *n* = 579			Adolescents *n* = 290			Adults *n* = 289		
	Total fluid intake (mL/day)	Number of drinking acts (*n*)	Volume/act (mL)	Total fluid intake (mL/day)	Number of drinking acts (*n*)	Volume/act (mL)	Total fluid intake (mL/day)	Number of drinking acts (*n*)	Volume/act (mL)
24-h dietary recall	2073	6.7	320	1982	6.3	326	2164	7.1	314
7-day fluid record	2454	8.9	280	2390	8.4	289	2520	9.3	273
Difference (24 h recall − 7-day records)	−382 (−299, −465)	−2.2 (−1.9, −2.4)	39 (31, 47)	−408 (−298, −519)	−2.1 (−1.8, −2.5)	37 (25, 49)	−355 (−230, −480)	−2 (−1.8, −2.6)	41 (30, 52)
*p* value	<.0001	<.0001	<.0001	<.0001	<.0001	<.0001	<.0001	<.0001	<.0001
Spearman correlation coefficient (r)	0.333	0.201	0.477	0.326	0.229	0.492	0.279	0.129	0.457
*p* value	<.0001	<.0001	<.0001	<.0001	<.0001	<.0001	<.0001	0.0295	<.0001

Data are presented as mean (95 % CI) and tested with Wilcoxon rank test

**Fig. 4:**
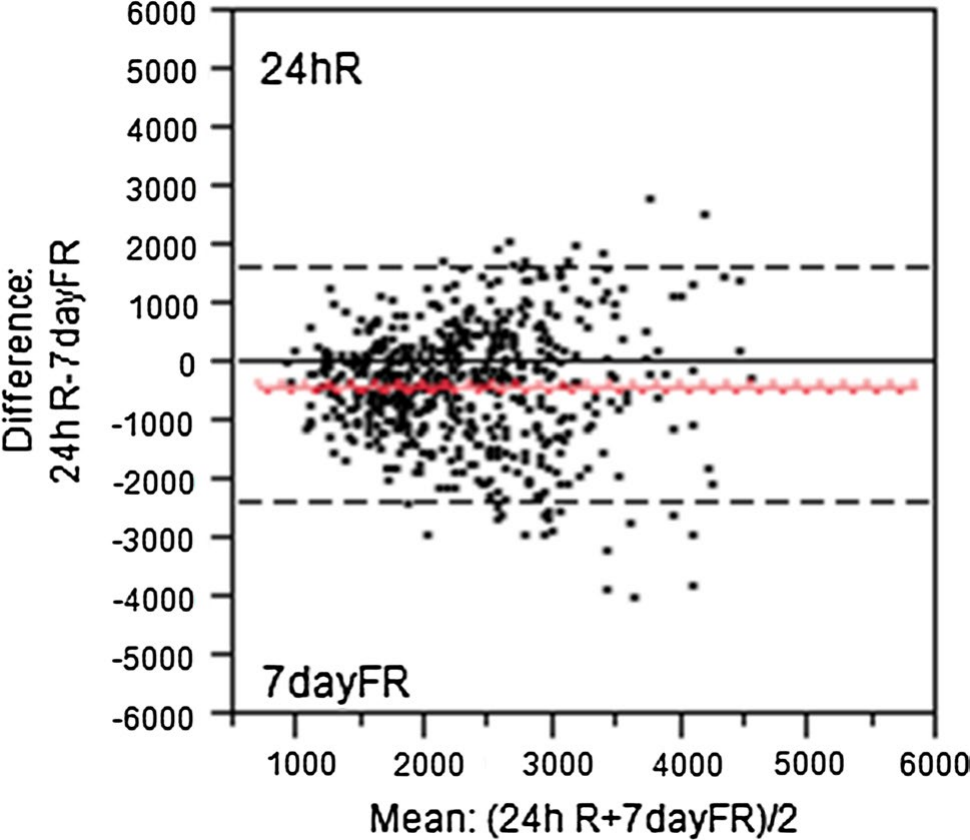
Bland–Altman plots to assess agreement between the 24-h dietary recall (24hR) and the 7-day fluid record (FR) for total fluid intake in the total sample. Each *dot* represents one subject participating in the study. The *red line* represents the mean difference (bias), and the dotted lines represent ±2SD from the mean (limits of agreement). The *solid line* is equal to 0, which indicates absolute agreement between 24hR and FR

A Bland–Altman plot for mean TFI recorded with the 24-h dietary recall and 7-day fluid record is presented in Fig. 4. Poor agreement between the two methods was observed as 11 % of intakes were outside of the limits of agreement. Moreover, Fig. 4 shows that the mean difference in TFI between both instruments increased with higher mean TFI. The total sample was therefore divided into quartiles based on mean TFI obtained with the 7-day fluid diary, and the mean difference in TFI recorded with the two instruments was calculated per quartile. For the lowest quartile of TFI, TFI was higher with the 24-h recall (+139 mL/day) than with the 7-day record. For subjects in the highest quartile of TFI, the mean difference in TFI increased to −1265 mL/day, with the 24-h recall underestimating the TFI compared with the 7-day fluid record.

The Spearman rank correlation test showed a significant correlation between the fluid intake data estimated with the 7-day consecutive fluid record and the 24-h dietary recall (*r* = 0.333; *p* < 0.001) (Table 3). This correlation was independent of age group (adolescents *r* = 0.326; *p* < 0.001; adults *r* = 0.279; *p* < 0.001).

Table 4 reports the proportion of subjects adhering to the Indonesian recommendation on water intake. The cross-classification of the adequacy percentage categories of mean TFI estimated with both instruments revealed that 53 % of subjects in the total sample were classified into categories of exact agreement; 83 % of subjects were classified into categories of exact agreement plus adjacent. The percentage of subjects classified into categories of disagreement and extreme disagreement were 13 and 4 %, respectively.

**Table 4:**
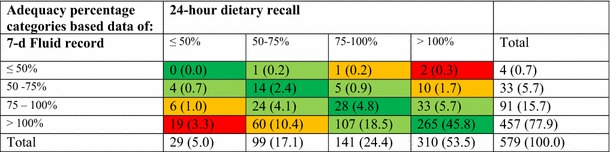
Proportion of subjects by adequacy percentage categories achieving the Indonesian recommendation on fluid intake (80 % of the recommendation of total water intake), based on total fluid intake estimated with a 24-h dietary recall and a 7-day fluid record

Data presented as *n* (%). Cells were colored dark green if the classification between instruments was in exact agreement, light green if classified into adjacent category, orange if classified two categories apart (disagreement between both instruments), and red if classified into extreme categories (extreme disagreement between instruments)

## Discussion

The aim of this study was to compare habitual TFI assessed with a 7-day fluid-specific record and a 24-h dietary recall among apparently healthy Indonesian adolescents (16–18 years) and adults (19–64 years). The results indicated that, at group level, the 24-h dietary recall significantly underestimated TFI compared with the 7-day fluid record, and that there was poor agreement between both methods.

There are several possible explanations for the differences in TFI at group level estimated with the two methods. Firstly, the 7-day fluid record is a prospective method, whereas a 24-h dietary recall is a retrospective method that depends on respondents’ memory, recall, and conceptualization abilities. The underestimation by the 24-h recall compared with the 7-day fluid diary might thus be due to subjects not recalling some drinking acts. This hypothesis is supported by the current results showing two extra drinking acts with the 7-day fluid record compared with the 24-h recall. A second explanation could be linked to the specificity of the instruments: With the 24-h dietary recall, both foods and fluids were recorded; therefore, subjects might have focused on their food and fluid intake at meal time. Depending on the country, fluids are more frequently consumed outside of meals [11]; thus, drinking acts outside of meals may have been overlooked during the 24-h recall. However, it is not possible to verify this hypothesis because information was not collected on the intake occasion (within or outside meals) at which the two extra drinking acts were recorded with the 7-day fluid diary. A third possible explanation for the difference in TFI is the fact that the 7-day fluid record takes into account TFI across 7 days, whereas a 24-h recall does not. Given that no day-to-day variability was observed with the 7-day fluid diary, this is unlikely to be the cause for the difference in TFI of both methods. Moreover, as shown in Table 2, the difference in TFI between the 24-h recall and *any day* of the 7-day fluid record was comparable to the difference in TFI between the 24-h recall and the mean of the 7 days of the fluid record. This means that the intake of a group estimated by the 24-h recall cannot be used/interpreted as the intake on any given day. A last possible explanation for the difference in TFI between both instruments could be a social desirability bias. When performing the 24-h recall, the subject was face to face with a nutritionist and might have over- or underreported their TFI or intake of a certain fluid type (e.g., overestimated the intake of a “healthy” fluid and underestimated the intake of an “unhealthy” drink) to meet a socially desired health standard. The over- or underestimating of a certain fluid type was, however, considered to be unlikely as the subjects consumed 75–76 % of their TFI as water. Moreover, the contribution of the different fluid types to TFI was comparable between both methods. An over- or underreporting of the volume consumed was likely as the volume per drinking act significantly differed between both methods, with a higher volume per drink act observed with the 24-h recall. These observations suggest that (1) with a 24-h recall, subjects recall two drinking acts less than with a 7-day fluid record, but they estimated their volume consumed per drinking act to be larger and (2) a 24-h recall seems to be an acceptable method if the aim was to describe the contribution (in %) of different fluids to TFI of a group. The latter observation should, however, be confirmed in a sample with a more diverse fluid intake pattern than the one of this Indonesian sample (75–76 % of TFI was water).

Despite the disagreement in TFI measured with both instruments, the TFI data of the 24-h recall and TFI of 7-day fluid record were significantly correlated. Yet, according to the Dancey and Reidy’s [12] categorization, the correlations were weak to moderate. Additionally, a correlation coefficient measures the strength of a relation between two variables, not the agreement between them [10]. During the interpretation of the results, importance is therefore mainly given to the Bland–Altman method, which is recommended as the preferred method for the comparison of measurement or assessment methods [13].

Some limitations of the study need to be acknowledged. Since both instruments were compared in the absence of an objective biomarker of TFI, this is a relative comparison. In nutrition however, when different instruments are compared, a 7-day dietary record is frequently used as the reference method [6, 14]. Since the 7-day dietary record in this study was specifically focusing on fluids, the assumption was made that the accuracy and specificity for recording of TFI were even higher than the standard dietary (food and fluid) record. The high prevalence of obesity observed in the adult population might also be a limitation, since the adult sample might not be representative of the whole Indonesian adult population. It is known that obese subjects significantly underestimate intakes, and therefore, this could potentially have affected the results [15, 16]. However, the possible effect on reporting due to the high prevalence of obesity was minimized by the crossover study design, in which each subject is his own control. A third limitation is related to the habitual intake of different fluid types in Indonesia. The subjects included in this study, but also adolescents and adults participating in another survey in Indonesia [17, 18], appeared to consume mainly water. The other fluids contributed only a small amount to TFI. Therefore, differences in reporting of certain fluid types other than water might not be detectable in this study. Additionally, the statistical model did not account for periods, sequence, and clustering by community and household. However, the effect of period was controlled for by randomization at group level. No or minimal period effect (or carryover effect) was anticipated as subjects were requested to keep their habitual food and fluid intakes. A last limitation to acknowledge is that food intake was not controlled over the study period. Since food intake can influence fluid intake [19], the possibility remains that fluid intake at individual level differed by study week due to a different food intake. Subjects were, however, requested not to change their food intake during the study period.

This study also had several strengths. Firstly, a large sample was recruited, with an equal distribution of gender and an equal coverage of both age groups. Moreover, the study was completed with a high compliance rate (i.e., >80 %). The crossover approach is a second strength to highlight. This enabled a pairwise comparison between both instruments, but it also allows to conclude that the systematically higher fluid intake estimated with the 7-day fluid record is not due the nutritionists improving their interview skills or participants learning over time to report more fluid intake. A third strength worthy of mentioning is the use of the Bland–Altman method for data analysis, which is the most recommended validation procedure [10, 13]. This analysis can indicate whether two measurement techniques agree sufficiently to be interchanged [10]. The results of this study indicated that a 24-h dietary recall cannot replace a 7-day fluid diary.

The results of this study have implications in several contexts. The current recommendations on total water intake in Indonesia, but also in the USA defined by the Institute of Medicine, are adequate intakes based solely on median observed intakes [4, 20]. The dietary reference values for total water intake set by the European Food Safety Authority are also adequate intakes based on observed intakes, although other factors including achievable or desirable urine osmolality were also considered [21]. These observed intakes were derived from population surveys in which 24-h recall was used most frequently [22]. The current results suggest that the observed intakes and consequently also the recommended adequate intakes of total water might be underestimated. Additionally, the outcomes of an evaluation of adequacy of total water intake might differ substantially depending on the instrument used, as shown by the results of the cross-classification. The 24-h dietary recall indicated that 24 % of the subjects did not reach 75 % of the water recommendation, whereas the 7-day fluid record estimated this proportion to be 17 %. The implication of this different adequacy evaluation depends on the fluid type involved. In the case of water intake, a 24-h dietary recall might overestimate the number of individuals at risk for a negative health impact associated with a low water intake (e.g., chronic kidney disease, or new-onset hyperglycemia) [23, 24]. This, however, does not necessarily have negative consequences; an individual with a low water intake will be recommended to drink more, preferably water, and this has been shown to be beneficial for health [23, 25, 26]. In the case of other beverages such as soft drinks, a 24-h recall may overestimate the number of individuals with a low consumption or underestimate the number of individuals with a high consumption. Consequently, the size of the risks associated with an excessive soft drink consumption such as weight gain [27], development of metabolic syndrome [28], type 2 diabetes [29], or other health problems [30] may be underestimated by surveys using 24-h recalls.

## Conclusion

When compared with a 7-day fluid record, a 24-h dietary recall significantly underestimated TFI with the difference between TFI assessed with these two instruments increased with increasing mean TFI. Both instruments showed disagreement in 23 % of the study population on the classification of the adequacy of their water intake. These results suggest that the evaluation of the public health risk associated with inadequate or over consumption of different types of fluids based on 24-h recall data may not be accurate, and that the adequate intake for total water (currently based on 24-h intakes) may require revision in the future.
